# Ethical issues related to research on genome editing in human embryos

**DOI:** 10.1016/j.csbj.2020.03.014

**Published:** 2020-03-21

**Authors:** Emilia Niemiec, Heidi Carmen Howard

**Affiliations:** Centre for Research Ethics and Bioethics, Uppsala University, Box 564, 751 22 Uppsala, Sweden

**Keywords:** Genome editing, CRISPR-Cas9, Oocyte donation, Egg donation, Whole genome sequencing, Research ethics

## Abstract

Although the potential advantages of clinical germline genome editing (GGE) over currently available methods are limited, the implementation of GGE in the clinic has been proposed and discussed. Ethical issues related to such an application have been extensively debated, meanwhile, seemingly less attention has been paid to ethical implications of studies which would have to be conducted in order to evaluate potential clinical uses of GGE.

In this article, we first provide an overview of the debate on potential clinical uses of GGE. Then, we discuss questions and ethical issues related to the studies relevant to evaluation of potential clinical uses of GGE. In particular, we describe the problems related to the acceptable safety threshold, current technical hurdles in human GGE, the destruction of human embryos used in the experiments, involvement of egg donors, and genomic sequencing performed on the samples of the research participants.

The technical and ethical problems related to studies on GGE should be acknowledged and carefully considered in the process of deciding to apply technology in such a way that will provide benefits and minimize harms.

## 1 Introduction

The CRISPR-Cas9 genome editing (GE) system allows for precise, efficient, relatively cheap, and fast modification of DNA in various organisms and types of cells. GE has been found to have applications in many research areas, including, among others, gene function studies, gene therapy studies, drug development, and production of modified crops in agriculture [1]. In 2015, the first experiment with CRISPR-Cas9 on human embryos was reported; the authors demonstrated that the GE system performed targeted cleavage in the β-globin gene, which when mutated causes β-thalassemia [2]. Following this publication, discussion on prospective clinical applications of germline genome editing (GGE) in humans and related ethical issues was ignited. While the term GGE refers to applications of GE on progenitor cells of gametes, gametes, or embryos, in this article, we focus our reflection on GGE conducted on human embryos.

In spite of legal prohibitions on germline genome modification to establish a pregnancy (i.e. what we consider a clinical use) enacted in many countries [3], clinical uses of GGE have been discussed by prominent scientists [4], [64]. Many meetings and groups have been convened to address the ethical questions surrounding the technique and a voluminous academic literature on this topic has quickly grown. The majority of the scientific community currently considers the clinical applications of GGE premature and many groups have called for a moratorium for such uses [5], [6]. Nevertheless, future implementation of GGE in the clinic has not been excluded as a possibility. Many policy documents, professional recommendations, and groups of authors either state or seem to imply that GGE in the clinic could be acceptable if certain conditions are met. Requirements specified most often include adequate safety and efficacy of the method, societal debate and/or societal consensus, and appropriate governance [6], [7], [8], [9], [10], [11].

Benefits, risks, and ethical issues of potential clinical uses of GGE have been discussed at length, meanwhile, seemingly less attention has been given to the questions raised by research on human GGE which would have to be conducted in order to address safety and efficacy of the technique. In this article we offer an analysis of ethical issues related to the research context of GGE, including interrelated conceptual and technical aspects. To place this analysis in a broader context, we first present the discussion on potential clinical uses of GGE in humans focusing on claimed benefits, risks and selected ethical issues. We then tackle the questions related specifically to research on human GGE concerning: challenges in the evaluation of safety and efficacy; technical shortcomings and the safety risks they pose; problems related to use of embryos in research; oocyte procurement; and genomic sequencing.

## 2 Germline genome editing in the clinic: potential benefits, risks and ethical issues

Although claims of the potential benefits of GGE often relate to the possibilities of curing or preventing disease (see for example Gyngell, 2017 [12]), the actual advantage of GGE over available methods is uncertain and currently appears limited. Firstly, GGE does not have curative aims *per se* since there is *no patient* involved in the procedure who could be cured^1^ [13]. Instead, GGE in the clinic would be coupled with the in vitro fertilization (IVF) technique in order to *create* a genetically-related child (for a given couple) who would then possess a desired trait (e.g. would not be affected by a given disease).

This approach would be offered in the first instance to couples who have a combination of genotypes that will result (at least in theory) in some of their children being affected by a genetic disease and who are aware of this and would like to avoid passing the disease to their offspring. Importantly, such couples currently have a number of options available to achieve the desired goal: 1) undergo IVF coupled with preimplantation genetic diagnosis (PGD) to select “unaffected” embryos and use them to establish pregnancy; PGD can detect post-IVF embryos which carry disease-causing alleles and allows for the selection of embryos without these (combinations of) alleles; 2) use donor gametes in IVF; 3) adopt a child; 4) decide to not have a child together.

In the majority of cases, IVF coupled with PGD can be applied^2^ and GGE does not seem to have a clear advantage over it, as we explain in the paragraph below. Yet, at least in theory, there are rare cases where all children of a couple would be affected by a disease (for example, when one parent is homozygous for a dominant disease, or both parents are homozygous for a recessive mutation) and there is no option for PGD and embryo selection. In such situations, GGE might potentially be the only alternative to have a genetically related child not affected by a disease, and, as such, it could bring an advantage over currently available methods [14]. Importantly, it is not clear whether such couples exist and if they do exist, whether they would be willing to undergo GGE. Indeed, the theoretical estimation based on available data on prevalence of genetic disorders in the USA suggests that the clinical demand for GGE would be very small [15]. For example, the analysis indicates that in the USA in a given time there is only one couple at reproductive age in which both persons are homozygous for variants causing cystic fibrosis [15].

Some authors have suggested another benefit of GGE, namely the possibility to *rescue embryos* already created in IVF, where disease-causing variants are detected by PGD and which would normally be discarded [16], [17]. As argued by these authors, such embryos could undergo GGE and subsequently be transferred *in utero* to establish a pregnancy; this, as the authors claim, would rescue embryos and increase efficiency of the procedure of IVF. This view of “rescuing” embryos is, however, challenged by technical hurdles, for example, the fact that GGE seems to be best applied at the moment of fertilization to prevent mosaicism, which precludes the possibility of testing and identifying embryos with a disease-causing gene variant [14], [15].

Another suggested group of applications of GGE has, as a goal, the general enhancement of (often complex) traits and an increased resistance to diseases (the latter often regarded as a subset of enhancement, namely disease-prevention enhancement). The proposals to enhance traits such as intelligence and other complex traits are currently premature due to scientific and technical limitations. These are primarily related to an incomplete knowledge about the (often limited) genetic contributions to complex traits, as well as difficulties related to the more complicated approach that would have to be employed to edit multiple gene variants. Regarding the suggestion to increase resistance to a disease, controversially, the first reported case of *clinical* GGE represents such an attempt. In November 2018, He Jiankui, a scientist at the Southern University of Science and Technology in Shenzhen (China) showed results of his clinical study in which he edited genomes of human embryos that were subsequently used to establish a pregnancy [18]. He claims that two baby girls with edited genomes were born as a result of this experiment. The goal of this GGE clinical study was to modify the gene *CCR5* with an aim to increase resistance to HIV in children whose biological father was affected by AIDS. The study of He Jiankui has been widely criticized not only due to a lack of medical need justifying the research and the presence of alternative measures to avoid contraction of HIV in that situation, but also due to violation of a number of ethical requirements including not seeking and gaining ethical approval of the study and not ensuring that adequate informed consent was obtained [18].

Potential benefits of GGE may be discussed, as argued by some authors, from a broader perspective which includes long-term benefits on a population level such as a decrease in prevalence of genetic diseases [19]. Importantly, such a prognosis assumes not only that the technique will be efficient and safe, but also that its uptake will be high, neither of which can be taken for granted. Even if the prospect of reducing the frequency of diseases in a population was realistic, such an advantage and related economic gains should be weighed against harm which may be caused to individuals, on which we elaborate below.

Importantly, in both types of GGE applications (to obtain a child without a disease-causing gene and for enhancement), the approach would be used to satisfy a desire to have a genetically-related child with a chosen feature. It is important to acknowledge that as such, GGE does not fulfill a therapeutic purpose *per se*, since the technique is being used to create the child (with a specific trait) and not to treat an already existing child. As such, the rationale of fulfilling the desire for a biologically related child with trait “X” funded by (public) healthcare services can be challenged, as has been done with other reproductive technologies [20].

Furthermore, GGE in the clinic poses many additional ethical problems and risks. First, proceeding with first-in-human uses of GGE, unlike in the case of other “standard” clinical trials, will involve *creating a research participant,* who would be involuntarily involved in a research project. Importantly, the main expected benefit of such a trial would be, as already mentioned, satisfying the parents' desire to have a genetically-related child not affected by a given disease. To achieve this goal, a child would be involved in an experiment with relatively high uncertainties, risks, and potentially irreversible consequences. Furthermore, the experimentation and consequent need for monitoring may result in such a person (as well as their children and grandchildren etc.) taking part in a life-long experiment or clinical trial essentially without having given consent. Such a situation raises fundamental questions about the autonomy and best interest of a child. Smolenski (2015) suggests that a decision to place a child in such a situation is not similar to other decisions parents routinely make for their children (e.g. because it is irreversible) and may extend the limits of parental liberty and decision making over a child to unacceptable levels [21].

Additionally, there are questions about the impact of such an intervention on child-parent relationships. In particular, the risk of reducing these relationships “*to an overt commercial transaction*” has been discussed [22], as well as the risk of making “*parents less tolerant of perceived imperfections or differences within their families”,* and of eroding *“parental instincts for unconditional acceptance.”* [9] Moreover, the efforts to obtain a child with desired traits, raise concerns about the rise of consumer eugenics “*in which affluent parents seek to choose socially preferred qualities for their children*” [23]. An increased focus on avoiding certain traits and increasing others may also lead to a decrease of compassion and general acceptance of disabled persons in society.

While not specific to GGE, ethical concerns about the creation and destruction of supernumerary embryos created during IVF but not used to establish a pregnancy may be also debated. We discuss ethical positions on the moral status of embryos in the section below.

## 3 Current guidelines on the potential use of GGE in the clinic: how do these impact research?

The theoretical or potential benefits of GGE in the clinic currently appear limited and uncertain (especially given the availability of alternative approaches), while the potential risks including the ethical problems it raises are numerous. There are authors who have been explicit about their view that GGE should not be pursued [24], [25]. There seems to be, however, many recommendations issued by professional organizations, policy-making groups, and other groups of authors (not representing any one group) that state that GGE is not currently permissible, but that such applications *might* be permissible provided certain conditions are met [6], [7], [8], [9], [10], [11].

What conditions should be met to pursue the (currently theoretical) limited benefits of GGE in the clinic according to the documents mentioned above? Most common recommendations refer to the need to address the current uncertainties surrounding the science, especially safety and efficacy concerns, and consequently to continue research on GGE [7], [8], [9], [10], [11]. The other, often expressed conditions are of societal debate and/or societal consensus on the issues of clinical GGE and appropriate oversight [6], [7], [8], [9], [10], [11].

In the remainder of this article we focus on the research context of GGE, as it is being supported, especially as stated above to address further uncertainties surrounding the science of GGE and especially its safety and efficacy (which we consider herein as including the basic science of how well genome editing tools are working on target edits).

## 4 Research context of GGE and ethical implications

### 4.1 Challenges related to the evaluation of safety and efficacy of GGE

As mentioned above, many policy documents and recommendations issued by professional groups as well as by individual authors state that safety and efficacy of GGE must be further studied and evaluated in order to consider its potential implementation in the clinic. However, less attention has been given to trying to answer the question: what is an acceptable threshold of safety and efficacy to proceed with clinical trials? And, exactly what kind of studies should be conducted in order to provide satisfactory evidence? While we do not offer the answers to these questions, we show in this subsection how these aspects are particularly problematic for GGE research and how this is recognised by different professional and policy groups.

Importantly, the above-mentioned questions arise in a broader context of a benefit-risk evaluation, which should precede any decision on initiation of a clinical trial. The classic and widely accepted medical ethics document, the Declaration of Helsinki states:

*‘‘Medical research involving human subjects may only be conducted if the importance of the objective outweighs the risks and burdens to the research subjects. (…) All medical research involving human subjects must be preceded by careful assessment of predictable risks and burdens to the individuals and groups involved in the research in comparison with foreseeable benefits to them and to other individuals or groups affected by the condition under investigation.’’*[26]

In this specific context of GGE, a question may be posed: could the importance of the objective of the trial, that is to create a genetically related child not affected by a given condition ever outweigh and justify exposing a child to the risks and uncertainties involved in GGE? Or, in other words, should the desire to have a genetically-related child be satisfied in spite of the harm the process can cause?

Of note, a positive answer to this question has already been given in the context of other reproductive technologies such as IVF, PGD, and more recently, nuclear genome transfer (also known as a mitochondrial replacement), which are now used in the clinic. The acceptance and clinical uses of nuclear genome transfer, which is considered by some as the first germline genome modification in the clinic,^3^ may, to some extent, pave the way to the potential implementation of GGE in the clinic. In this context, it is important to recognize that these approaches raised safety concerns and ethical issues, which were deemed tolerable as clinical uses were allowed. Flaws in the process of policy-making on these technologies have been discussed by commentators. For example, with regard to nuclear genome transfer, Drabiak explains:

*"The HFEA Review acknowledged the potential for complications pertaining to safety and efficacy, but unilaterally disregarded what the scientific community has described as numerous substantial barriers."*[27]

These concerns should prompt us to increase vigilance over the processes leading to decisions on the uses of GGE.

The challenges around safety assessments pertain not only to the fact that GGE in the clinic would not be, strictly speaking, therapeutic and involve the creation of a child, but also to the problem of long-term impacts from the irreversible changes introduced, both on the developing organism of a “genome-modified” person as well as on her descendants. The American Society for Gene and Cell Therapy and the Japan Society of Gene Therapy address this issue with the following:

*‘‘The requirement that the results of an experiment be susceptible to analysis and characterization before further applications are undertaken cannot be met with human germ-line modification with current methods, because the results of any such manipulation could not be analyzed or understood for decades or generations—a situation incompatible with ethical imperatives and with the scientific method.’’*[28]

In spite of the open question of whether a thorough assessment of the technique is possible at all, efforts have been taken to provide some guidance regarding what evidence is needed. The American Society of Human Genetics (2017) specified that the

*“(…) minimum necessary developments should include the following:*

*-Definitions of broadly acceptable methodologies and minimum standards for measuring off-target mutagenesis.*

*-Consensus regarding the likely impact of, and maximum acceptable thresholds for, off-target mutations.*

*-Consensus regarding the types of acceptable genome edits with regard to their potential for unintended consequences.”*[9]

In the summer of 2019, the International Commission on the Clinical Use of Human Germline Genome Editing (convened by the UK's Royal Society and the US National Academies of Sciences and Medicine) issued a call for evidence, that is, a request directed to experts in the field of genome editing to answer questions such as

“*If there were to be an appropriate use case for human germline genome editing, what evidence would be needed to proceed to first in human use?*”

*“What is the status of the technology for validating that a correct edit (on target characterization) has been made and that unintended edits (e.g., off target effects, mosaicism, etc.) have not occurred in a range of cell and tissue types? If possible, please provide evidence drawn from work on early stage human embryos.”*[29]

The Commission has, as its main goal, the development of “*a framework for considering technical, scientific, medical, regulatory, and ethical requirements for germline genome editing, should society conclude such applications are acceptable*.” [58]. In addition, they list specific requirements (or tasks) to identify appropriate pre-clinical approaches to assess on- and off- target effects, mosaicism, and long-term side effects, among other thing [58].

In the next section, we aim to provide a general overview of the selected technical aspects of the studies which raise important ethical implications. Importantly, studies addressing safety and efficacy of human GGE may be performed on animals or human embryos. Although we do not dismiss the importance of addressing ethics of the former, due to the limited space herein we focus on the problems raised by research on human embryos.

### 4.2 Safety issues in germline genome editing

In Table 1 we list studies published in English that have used GGE in human embryos. Although the goals of the experiments vary, all these studies may, to varying degrees, provide information on the functioning of genome editing in embryos. We may distinguish research which alludes to GGE in the clinic more directly, that is, studies in which disease-causing variants have been corrected with GE [30], [31], [32] or a study in which an allele of the gene *CCR5* associated with a resistance or slower progression of HIV infections has been introduced [33]. There are also studies which aimed to show feasibility of a given approach, but unlike the previous group of experiments, they did not focus on correcting disease-causing genes or variants relevant to disease resistance [34], [35]. Furthermore, research examining the role of a gene in embryogenesis using GE has been published [36].

**Table 1:** Studies conducted using germline genome editing on human embryos. This table is based on Table 1 included in the article by Niemiec and Howard (2020) published under Creative Common Attribution Noncommercial License (https://creativecommons.org/licenses/by-nc/4.0/).

	Year	Authors	Title	Type of modification introduced	Type of embryos used
Clinic	2018	He Jiankui (unpublished, presented at the International Summit on Human Gene Editing, Hong Kong, 2018)	Developing a *CCR5*-targeted gene editing strategy for embryos using CRISPR/Cas9	Modification of *CCR5* gene to increase resistance to HIV infections	Embryos created with sperm of a man who contracted AIDS. Two embryos were implanted to establish a pregnancy which resulted in the live birth of twin girls
Research	2019	Li et al.	Efficient generation of pathogenic A-to-G mutations in human tripronuclear embryos via ABE-mediated base editing	Single nucleotide substitutions in a few genes (base editing)	Tripronuclear embryos created in clinical IVF procedures
	2019	Zhang et al.	Human cleaving embryos enable robust homozygotic nucleotide substitutions by base editors	Single nucleotide substitutions in a few genes (base editing)	Embryos created using immature oocytes from patients undergoing clinical IVF procedures and sperm from donors Tripronuclear embryos obtained in clinical IVF procedures
	2018	Zeng et al.	Correction of the Marfan syndrome pathogenic *FBN1* mutation by base editing in human cells and heterozygous embryos	Correction of a mutation in *FBN1* gene causing Marfan syndrome by base editing	Embryos created for the purpose of research using immature oocytes from women undergoing IVF procedures
	2017	Zhou et al.	Highly efficient base editing in human tripronuclear zygotes	Single nucleotide substitutions in a few genes (base editing)	Tripronuclear embryos
	2017	Li et al.	Highly efficient and precise base editing in discarded human tripronuclear embryos	Single nucleotide substitutions in a few genes (base editing)	Tripronuclear embryos created in clinical IVF procedures
	2017	Ma et al.	Correction of a pathogenic gene mutation in human embryos	Correction of a mutation that causes hypertrophic cardiomyopathy	Embryos created for the purpose of research (over 100 embryos were created) using oocytes and sperm procured specifically for research
	2017	Tang et al.	CRISPR/Cas9-mediated gene editing in human zygotes using Cas9 protein	Correction of a mutation in *HBB* gene causing β-thalassemia and a mutation in *G6PD* gene related to an enzyme deficiency	Embryos created for the purpose of research using immature oocytes and sperm from patients undergoing clinical IVF procedures Tripronuclear embryos created in clinical IVF procedures
	2017	Liang et al.	Correction of β-thalassemia mutant by base editor in human embryos	Correction of a mutation in the *HBB* gene which causes β-thalassemia (base editing)	Embryos obtained by somatic cell nuclear transfer; immature oocytes were donated by women undergoing IVF procedures
	2017	Fogarty et al.	Genome editing reveals a role for OCT4 in human embryogenesis	Study of the function of the pluripotency transcription factor OCT4 during embryogenesis	Surplus embryos created in clinical IVF procedures
	2016	Kang et al.	Introducing Precise Genetic Modifications into Human 3PN Embryos by CRISPR/Cas-Mediated Genome Editing.	Introduction of an allele of the gene *CCR5* associated with a resistance or slower progression of HIV infections	Tripronuclear embryos created in clinical IVF procedures
	2015	Liang et al.	CRISPR/Cas9-Mediated Gene Editing in Human Tripronuclear Zygotes	Modification of *HBB* gene, which when mutated causes β-thalassemia	Tripronuclear embryos created in clinical IVF procedures

The studies presented in Table 1 reveal, among other aspects, various technical hurdles, which render potential clinical applications of GGE unsafe. The main technical problems with safety implications for potential clinical GGE in human embryos revealed therein include:

1) mosaicism, a situation where not all cells of an embryo/organism have the same DNA - in this case the desired DNA modification;
2) off-target effects, where unintended changes in the genome outside of the targeted sequence occur;
3) on-target undesired modifications introduced within or next to the targeted locus [16].

All of these phenomena may have negative and difficult to predict effects on an organism. Some strategies exist to address these problems, albeit to varying degrees. For example, a study by Ma et al. (2017) has shown that injection of CRISPR-Cas9 system at the moment of fertilization reduces mosaicism [30]. Ma and colleagues also explicitly admitted technical shortcomings of their approach, in particular occurrence of on-target effects:

*“Despite remarkable targeting efficiency and high HDR [homology-directed repair] frequency, some CRISPR–Cas9-treated human embryos demonstrated NHEJ [non-homologous end joining] induced indels and thus would not be suitable for transfer. Therefore, genome editing approaches must be further optimized before clinical application of germline correction can be considered.”*[30]

An alternative GE approach has been developed and tested in human embryos, whereby, unlike in CRISPR-Cas9 system, sequence is not cut, but a base pair is directly modified [32], [34], [35]. As such, this base editing approach does not involve a repair mechanism which could introduce undesired on-target modifications. Yet, this technique is not only relatively new and not thoroughly tested, but also was shown to introduce off-target modifications [59], [60].

Not only does the elimination of these undesired events in the genome pose challenges, but so do the very techniques used to attempt to detect these changes. In order to control the effects of genome editing, an embryo has to be biopsied, DNA isolated and sequenced. Since the amount of isolated DNA in such context is relatively low, DNA has to be pre-amplified which introduces risks of errors [16]. Furthermore, there are problems with an adequate reference genome sequence to which the sequence of an edited embryo could be compared. The DNA sequence of an edited embryo can be compared to the parents’ sequences and reference genome (assembled, representative for humans whole genome sequence); in this case, however, potential off-target modifications have to be distinguished from other variations among genomes [16].

Besides the technical shortcomings revealed by the studies, there are also broader problems related to cell physiology and genomic interactions, which may be relevant to safety of potential clinical uses of GGE. For example, epigenetic effects may occur, which, as the American College of Medical Genetics and Genomics explains, “*may alter normal patterns of gene expression in some tissues*” [37]. Additionally, we may inquire about what would be the impact of a given DNA modification more broadly on the functioning of a given organism which may vary depending on, for instance, environmental factors. There are also questions about multiple (sometimes not yet discovered) functions of edited genes. Given the complexity of the human genome and the variability among individuals as well as epigenetic mechanisms (which are not yet completely understood), it is difficult or even impossible to predict all the consequences of GGE on an organism (and on future generations). Furthermore, the effects of each modification may be different, depending on what gene is edited, its function and location on the chromosome, as well as the type of editing used. Additionally, the impact of factors such as medium used to culture the embryos and the way of administrating the GE system may be considered.

Above we presented a general and non-exhaustive description of technical hurdles in GGE. Indeed, there are many nuances of methodological aspects, which may have impact on the technical outcome of GGE and its potential safety in humans, which we do not address here. To thoroughly evaluate the current state-of-art of GGE with regard to technical issues, safety, and efficacy, a systematic literature review should be conducted (of research conducted both in human cells and animals). Importantly, such a review can only be meaningful if so called “unsuccessful” experiments are also published and accessible [38]. In addition to technical shortcomings, there are questions about methodology and reproducibility of the studies.

Furthermore, the process of answering whether some technical deficiencies and uncertainties are acceptable in the pursuit of the potential limited (added) benefits of clinical GGE will involve different value judgments by stakeholders with a priori different values, priorities, and opinions. This brings us back to the more fundamental questions of whether safety issues can be fully addressed and what level of uncertainty we are able or willing to accept.

Notwithstanding the limitations of this overview, it is clear that there remain a number of important technical problems (and hence with safety) with GGE and currently there are no straightforward solutions to surmount them. We may expect that many studies would have to be conducted to address these issues, both on human embryos and animals. Below we attend to the ethical problems raised by the former group of experiments.

### 4.3 Use of embryos

The use of embryos in research, including the studies listed in Table 1, raises a number of ethical aspects. One commonly discussed ethical issue is that related to the destruction of human embryos. We can distinguish the following types of embryos used in GGE research based on their source:

1) so called supernumerary or surplus embryos, which are left over after clinical IVF procedures,
2) embryos created specifically for the purpose of research using gametes left over (surplus) from IVF,
3) embryos created specifically for the purpose of research using gametes procured specifically for research.

Furthermore, we may distinguish viable and non-viable embryos. The former term means that embryos are considered to be able to develop "normally" and result in a live birth when implanted into a woman’s uterus; non-viable embryos (e.g. tripronuclear embryos) are considered unable to develop "normally" and to result in a live birth if implanted into a woman’s uterus. Of note, from the scientific point of view, viable embryos are, in general, more advantageous than non-viable embryos, as the latter type possess abnormalities impacting their functioning.

Since human embryos are humans in the earliest developmental stage, their destruction raises ethical questions. While the full discussion behind different considerations of the human embryo is beyond the remit of this article, we can distinguish broadly, three main positions in discussions on the moral status/value^4^ of the human embryo:

a) human embryos have the same moral status as any other born human;
b) human embryos have some moral status/value, but not the same as a born human; there are variations within this view, for example, some say that moral status or value of embryos increases during their development;
c) human embryos have no moral status or their moral status/value is the same as of any other type of human cells.

If we assume the position that human embryos have the same moral status as persons, GGE experiments are considered unacceptable. Research on embryos is prohibited in some countries (e.g. Austria, Germany, Italy, Poland), however, the formulations of these legislations differ (https://hpscreg.eu/map). Furthermore, two important public agencies that fund research, the European Commission and National Institutes of Health in the USA, do not fund research projects involving the destruction of embryos [39], [40].

The second view on the moral status of embryos has been entrenched in the legislation of more countries whereby research on embryos is allowed with some restrictions (that is, some protection is granted to embryos) (see https://hpscreg.eu/map). An example, although debatable,^5^ of such protection is the so called 14-day rule stating that research can be conducted only until 14 days after fertilization; after this time, embryos have to be destroyed [41]. Furthermore, if assumed that human embryos have some moral status/value, the type of research they are used in and their number may be discussed, and conditioned, for example, upon the expected benefit of research. Moreover, some laws make a distinction between using embryos left over from IVF procedures and those created specifically for research. The Oviedo Convention, for instance, states that the creation of embryos specifically for research is not permissible [42].

In the studies on GGE, both viable and non-viable embryos have been used; in one study, by Ma et al. (2017) embryos were created specifically for the purpose of research. Notably, the usual number of embryos used in such procedures can be high. For instance, in the study conducted by Ma et al. (2017), over 100 human embryos were created and destroyed. The continuation of such experiments would certainly multiply these numbers. We believe that this ethical issue related to the implementation of GGE in the clinic, should be acknowledged and discussed, and society informed about it when public discussions are conducted.

### 4.4 Oocyte procurement

The study of Ma et al. (2017) showed that the strategy of administering CRISPR-Cas9 system at the point of fertilization is advantageous as it reduces the mosaicism in embryos [30]. To follow such a strategy, embryos have to be created specifically for research. Furthermore, since the authors aimed to “correct” a specific gene mutation, it was essential to obtain, and, in this case, create embryos with exactly this genotype. Creating embryos for GGE research raises questions about the source of gametes used, particularly human eggs. While supernumerary oocytes and sperm from IVF procedures can be used, there may be limited availability of gametes with desired genotypes. If a scientist would be interested to study embryos heterozygous for a given (disease-causing) gene, an alternative approach could consist of deriving sperm from an affected man and using wild types oocytes donated as surplus after IVF, as was done in the study of Zhang et al. (2019) [35]. Oocytes can also be procured from women specifically for research, which raises more profound ethical issues.

Within the studies on human GGE listed in Table 1, five experiments involved egg donation: in one study, oocytes were procured specifically for research [30], in the remaining studies, immature oocytes obtained in clinical IVF procedures were used [31], [32], [35], [61]. Although immature oocytes retrieved in IVF procedures are usually discarded, it has been shown that some of such oocytes can undergo in vitro maturation, be fertilized and develop into embryos and live births [62]. Consequently, it may be questioned whether all immature oocytes can be considered useless in the context of clinical IVF and whether women should be invited to donate such eggs. When donation of mature and "healthy" oocytes obtained during IVF process is considered, such questions seem even more pertinent. Ballantyne and de Lacey explain that:

*“Women having IVF have the option of fertilizing all the eggs retrieved and freezing spare embryos for future use. When women do not wish, for personal reasons, to freeze embryos, freezing spare eggs for fertilization and transfer in future attempts is a viable option. If the woman donates some of her eggs for research and her initial embryos fail to implant, she must undergo additional cycles of egg retrieval that may otherwise have been unnecessary. Although the personal costs of egg donation will differ for each individual woman, depending on her specific fertility problem, there are currently no cost-free eggs. Due to the uncertainties surrounding egg fertilization, embryo implantation, successful pregnancy, and the desire for future children; infertile women always are being asked to donate a potentially valuable resource. Therefore, it is not the case that women are being asked to donate “spare” or “surplus” eggs. Rather, they are being asked to donate eggs that are a potentially valuable personal resource that has typically required significant investment of time, money, discomfort, and anxiety to produce.”*[43]

Egg donation specifically for the purpose of research raises additional concerns. Oocyte procurement is a physically invasive procedure, which involves ovarian suppression, followed by ovarian stimulation and a surgical procedure of oocyte retrieval. The whole process involves not only many inconveniences, but also risks to the physical health or even the life of the woman involved. Discomforts include frequent visits to doctor’s office, injections of medicines, and undergoing a surgical procedure under sedation. Most frequent side effects include nausea, irritability and headaches, among others [63]. Ovarian hyperstimulation syndrome is a rarer, yet more serious complication, which may, in the worst-case scenario, result in death [44]. Notably, in the consent forms used in the study by Ma et al. (2017) death was mentioned three times in the context of different procedures^6^. Additionally, Schneider et al. have drawn attention to long-term potential risks for oocyte donors, such as breast cancer, which has not yet been adequately studied [45].

The question of whether experiments involving the procedure of oocyte retrieval are ethically acceptable is not new and has been discussed in the context of stem cell research. Magnus and Cho highlight the disproportion between the risks involved and potential benefits in this context:

*“These women are not pursuing the procedure for any reproductive or medical benefit to themselves; rather, they are exposing themselves to risk entirely for the benefit of others. If we were to think of them as simply clinical patients, their physician’s fiduciary obligations would seem to require counsel against undergoing such a procedure for no benefit.”*[46]

Importantly, in the case of GGE, which is strictly speaking not a therapeutic procedure, as explained above, the ratio of benefits to risks is even more difficult to accept.

Despite serious arguments against studies involving oocyte retrieval, such research on stem cells has been conducted and accepted by professional societies as permissible [47], [48] raising other sets of questions on *how* women should be engaged in such studies. In particular, a key issue is on ensuring that consent to research is informed and women are adequately compensated for the risks and inconveniences to which they are exposed. Sums of a few thousand dollars in compensation (in the study of Ma et al. (2017) of 5000 US dollars) do not seem inflated when we consider the serious risks involved in egg procurement. On the other hand, such amounts of money may constitute undue inducement to some women who are suffering various degrees of financial hardship or socio-economic disadvantage. Indeed, it may be difficult to avoid undue inducement and at the same time offer fair compensation.

As explained above, studies involving the creation of human embryos seem to be currently advantageous over other approaches from scientific point of view. Until now (to our knowledge) only one study involving embryo creation and egg donation for purpose of research has been performed to study GE, yet, if the goal of addressing safety and efficacy of GGE is to be pursued, we may expect more of such studies. This will entail exposing many women to risks. We believe that these aspects should be recognized and acknowledged in the discussions on GGE.

### 4.5 Genomic sequencing

As mentioned above, in order to verify whether an embryo has been edited in the desired way and to assess for off-target events, genome sequencing of embryonic cells is conducted. The entire genome of gamete donors is also sequenced (e.g. from blood) in order to act as a reference sequence. In these ways, researchers also obtain a lot of genomic sequencing information from gamete donors. One may ask if gamete donors are aware that all (or a large part of) their DNA will be sequenced and know the implications of this fact. Indeed, our recent study of informed consent forms used in the study of Ma et al. (2017) shows that genomic sequencing has not been explicitly mentioned in the forms, which raises questions about whether research participants were adequately informed about this important aspect of the research [49].

Importantly, the use of whole genome sequencing (WGS) and whole exome sequencing (WES) of research subjects in the "regular" (i.e. non GGE) genomic research context already raises important ethical, legal and social issues (ELSI). These ELSI commonly revolve around issues of privacy and confidentiality of the genomic data; how to obtain fully informed consent from research subjects; the possibility of subjects to withdraw from research; as well as issues regarding the return of research results, including the right not to know. While going into depth into each of these issues is beyond the scope of this article (see Pinxten and Howard 2014 for a review) [50], we provide an example of the complexity of some ELSI by explaining the challenges of obtaining informed consent below. We also highlight that in general, the ELSI surrounding the use of WGS and WES in research is still being debated and the logistics and policies needed to be implemented to offer responsible genomic sequencing are still being developed. Hence the use of an already ethically challenging approach such as genomic sequencing within the highly ethically contentious context of GGE only serves to exacerbate the ELSI, especially where gamete donors are concerned.

The difficulties related to obtaining truly informed consent for genomic sequencing (even outside of the context of GGE) have been discussed [51]. A requirement for informed consent is communicating relevant possible risks and benefits to research subjects. With genome sequencing, the amount of data generated, and the different results that could be obtained above and beyond the results for the initial reason to sequence, as well as the uncertainties still surrounding sequencing [52] are so great that it is not obvious that all this information can be transmitted explicitly and meaningfully during the informed consent process. For example, data about family relations, or about DNA variants that may cause susceptibility to other diseases (other than the one that triggered the initial need for sequencing) may be revealed by the sequence information. Questions then arise about which types of information should be returned to research participants, how it would be done and by whom? Ideally, the consent process should include all these possibilities, but ironically this is likely to result in very long consent forms, the length and volume of information alone sometimes contributing to confusion and lack of understanding. Consent procedures should also address the security of data storage and for how long data will be kept and if it will be used for secondary purposes or shared with any third parties nationally or internationally. Clearly this is a lot of information and providing it in a way that truly supports decision making (instead of simply being an exchange and signature of forms) is a challenge.

### 4.6 Other issues related to research on GGE

In addition to the issues described above, there are other concerns related to the research which would have to be conducted to introduce GGE to the clinic. As mentioned earlier, in addition to the studies on human embryos, research on animals would be necessary to assess the impact of embryonic DNA modifications on the development and functioning of an adult organism and future generations. Such research, similarly to the studies conducted in humans would likely involve oocyte retrieval, in vitro fertilization, and implantation of the fertilized eggs to establish pregnancy; these procedures may cause pain and distress to animals. Although the problem of harm caused to animals in research is neither new nor unique for the studies on GGE, the question of whether the objective of the study can justify the harm caused to animals seems to be particular in the context of GGE given the problems its potential clinical use entails.

Research on animals is widely (yet not universally) accepted if the experiments are scientifically justified and follow the framework of three “Rs” relating to the reduction of the number of animals in the experiments, refinement of the protocols (so that animals’ suffering is minimized) and replacement with other approaches not involving animals where possible (see, for example, the Directive of the European Union [53]). Each study involving GGE in animals will have to follow the local legislation and guidelines for this kind of research.

Another ethical aspect related to GGE research is the opportunity costs of funding this type of research instead of other types, as Baylis puts it in her recent book:

*“What other valuable research is not being done as a result of this investment? (…) what other medical needs are being underfunded?”*[54]

We could also ask, more concretely, whether research efforts, talents, and funds should not be directed to the studies investigating somatic GE, which does not involve so many contentious ethical aspects as GGE, yet, is a promising approach to treat or even cure a number of genetic diseases [55].

## 5 Conclusions

Despite the limited and uncertain medical need for clinical GGE, numerous ethical issues and risks related to such an application, and current prohibitions of germline genome modification in many countries, uses of GGE have been proposed and discussed. Furthermore, in some circles, it seems that the focus of the debate has recently shifted from the question of *if* GGE should be introduced to the clinic to *how* it should be done [56], [57]. Indeed, influential actors such as the US National Academy of Sciences have undertaken efforts to establish a framework for a potential translational pathway for clinical GGE [58].

Not only does the potential clinical use of GGE raise ethical issues, but so does the research context of this approach, including all the studies that would be required to evaluate GGE before its potential introduction into the clinic. Firstly, it can be questioned whether safety of the procedure could ever be sufficiently evaluated in the non-clinical studies since the effects of GGE on a developing human organism cannot be fully predicted. Related to this, there are questions about the degree to which numerous technical and scientific hurdles would have to be addressed and the type of evidence needed from the studies both on human embryos and animals. GGE research using embryos raises questions about the moral status of the human embryo, the involvement of egg donation, which entails serious risks to women, as well as the ethical issues related to whole genome sequencing. To evaluate the effects of germline DNA modification on a developing and adult organism as well as on future generations, research on animals would have to be involved, which raises ethical concerns as well. Last but not least, we may question whether continuation of such research is the best allocation of the resources, both in terms of funds and personnel.

We argue that these additional “costs” of bringing GGE to the clinic, related to the research context, should be acknowledged and carefully considered along with the potential benefits when evaluating further research and the potential clinical applications of GGE.

## CRediT authorship contribution statement

**Emilia Niemiec:** Conceptualization, Investigation, Writing - original draft. **Heidi Carmen Howard:** Conceptualization, Investigation, Writing - original draft, Supervision.

## Declaration of Competing Interest

The authors declare that they have no known competing financial interests or personal relationships that could have appeared to influence the work reported in this paper.

## Footnotes

1 In one of the approaches of GGE, the embryo can be considered as the subject which could be cured, that is, when GGE is applied on an existing embryo. This approach, however, is known to cause mosaicism in embryos, and as such, is unlikely to be seriously considered for clinical uses. The method of adding the components of a GE system at the moment of fertilization seems more advantageous in terms of safety of potential clinical uses.

2 The risks and impact of PGD on the human organism are more understood than those of GGE, since PGD has been used in the clinic for nearly 30 years. Yet, PGD raises ethical issues as well, many of which are common with GGE, for example, the questions related to the potential impacts on family relationships and more broadly on the whole society (see the discussion below).

3 While technically, nuclear genome transfer does imply a change in the genome since there is no longer the same combination of nuclear DNA and mitochondrial DNA – lumping both genome editing of specific nucleotides with the much coarser exchange of mitochondria (and cytoplasm) is somewhat misleading. Indeed, these two proceedures do not work at all on the same scale or entail the same type of changes at the DNA level and consequently the same types of risks and benefits. It has been suggested that by grouping them in the same category, the acceptance of one will open the door to the acceptance of the other.

4 For an overview of arguments related to moral status/value of human embryos, see for example, the report of the Nuffiled Council on Bioethics on the related topic [41].

5 It may be argued that 14-days rule protects more developed embryos from use and destruction in research, however, it does so by requesting destruction of less developed embryos.

6 The forms were provided at our request by one of the co-authors of the study of Ma et al. (2017). To our knowledge they are not available online.
